# Supplementary material for: Non-KREEP origin for Chang’e-5 basalts in the Procellarum KREEP Terrane
Source: Nature. 2021 Oct 19;600(7887):59–63. doi: 10.1038/s41586-021-04119-5 (PMC8636255; doi:10.1038/s41586-021-04119-5)
Supplement: Supplementary file 1 — This file contains Supplementary Figs. 1–3, showing the positions of analytical spots of trace elements and Sr–Nd isotopes for Chang’e-5 basalt clasts. [file 41586_2021_4119_MOESM1_ESM.pdf]

nature portfolio

https://doi.org/10.1038/s41586-021-04119-5

Supplementary information

Non-KREEP origin for Chang'e-5 basalts in the Procellarum KREEP Terrane

In the format provided by the authors and unedited

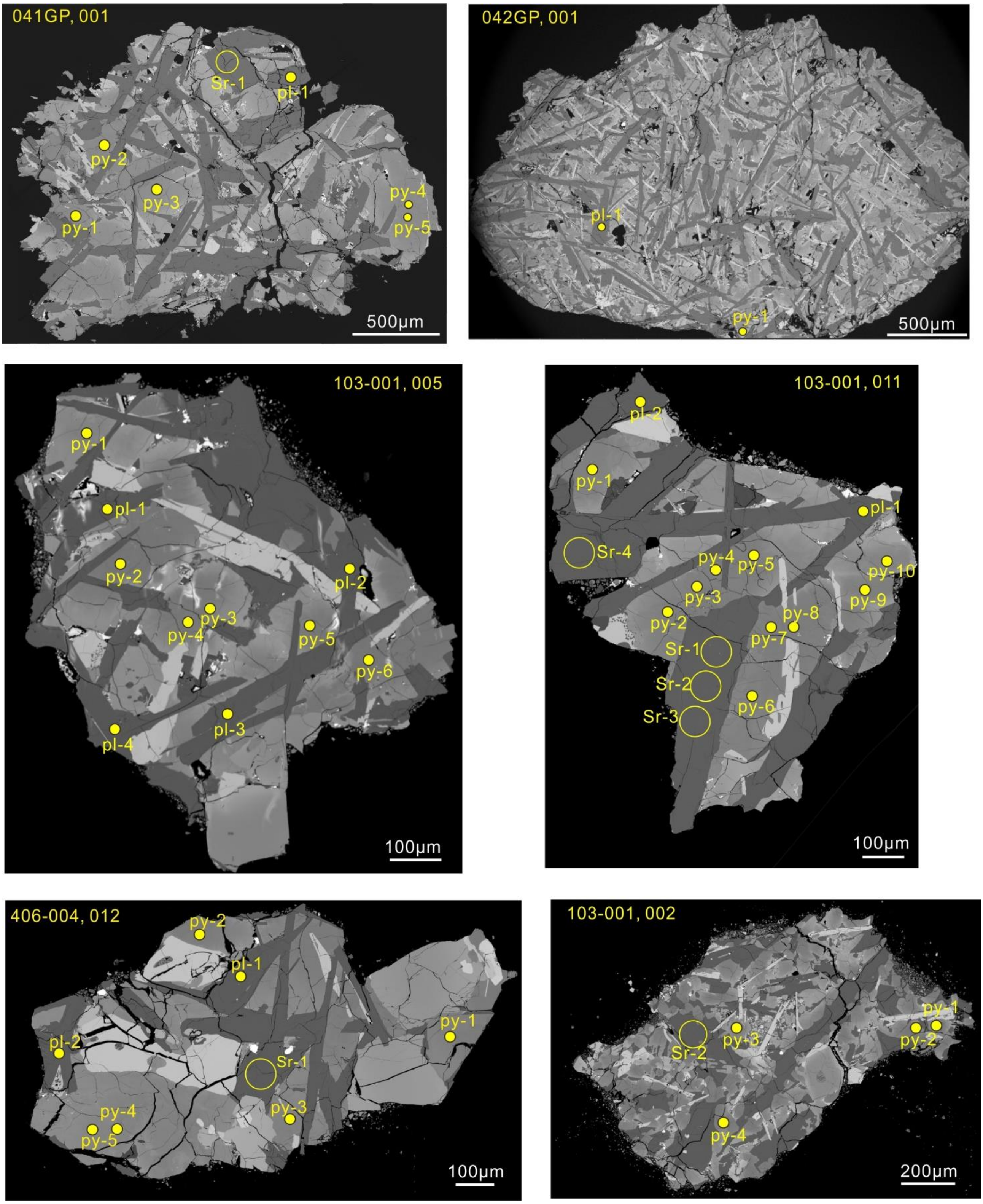

Figure S1. Backscatter Electron (BSE) images of the Chang'E-5 basalts (041GP,001; 042GP,001; 103-001,005; 103-001,011; 406-004,012; 103-001,002). The black circles filled with yellow color and yellow circles show the LA-(MC)-ICP-MS spots for trace elements and Sr isotopes, respectively.

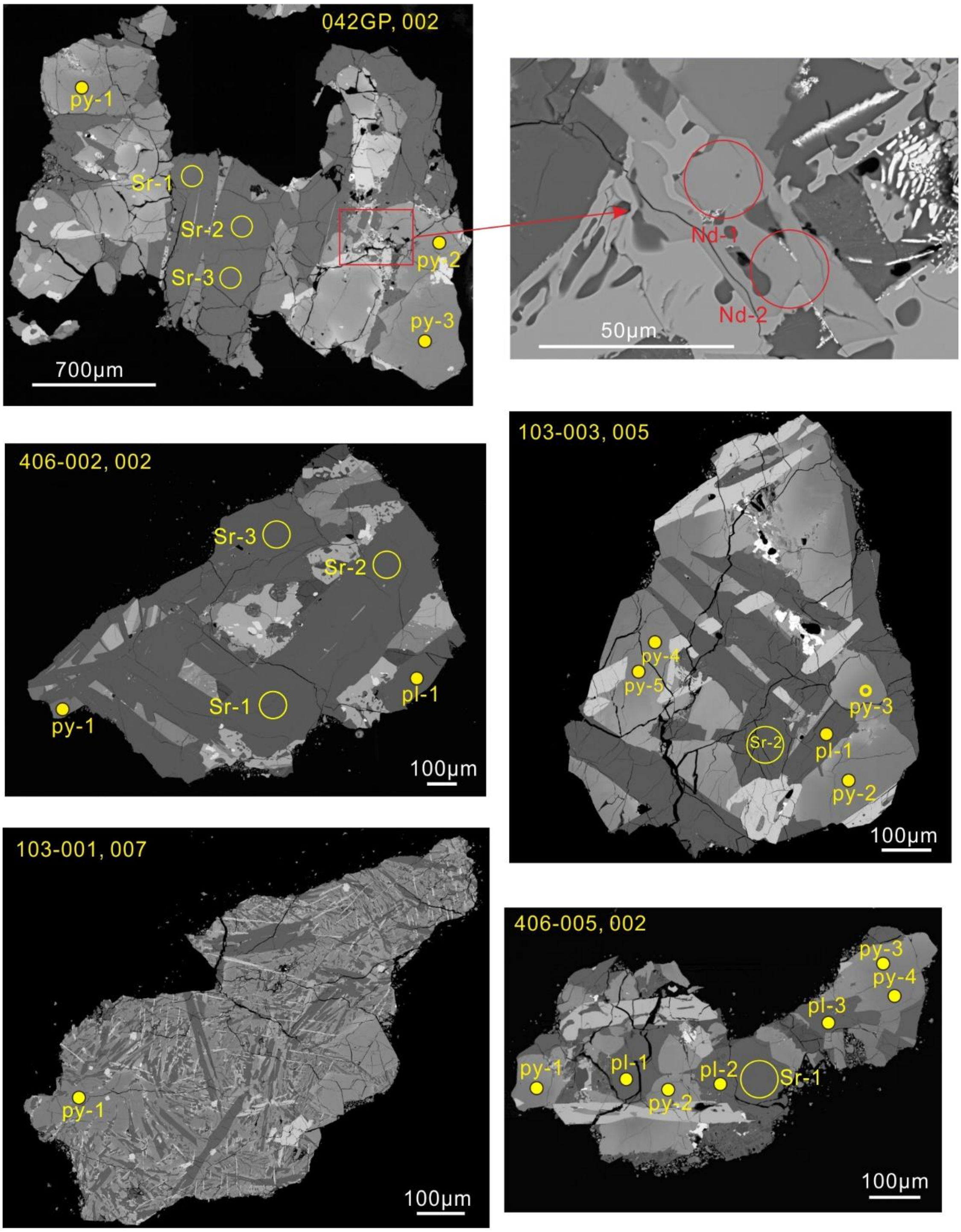

Figure S2. Backscatter Electron (BSE) images of the Chang'E-5 basalts (042GP,002; 406-002,002; 103-003,005; 103-001,007; 406-005,002). The black circles filled with yellow color, yellow circles, and red circles show the LA-(MC)-ICP-MS spots for trace elements, Sr, and Nd isotopes, respectively.

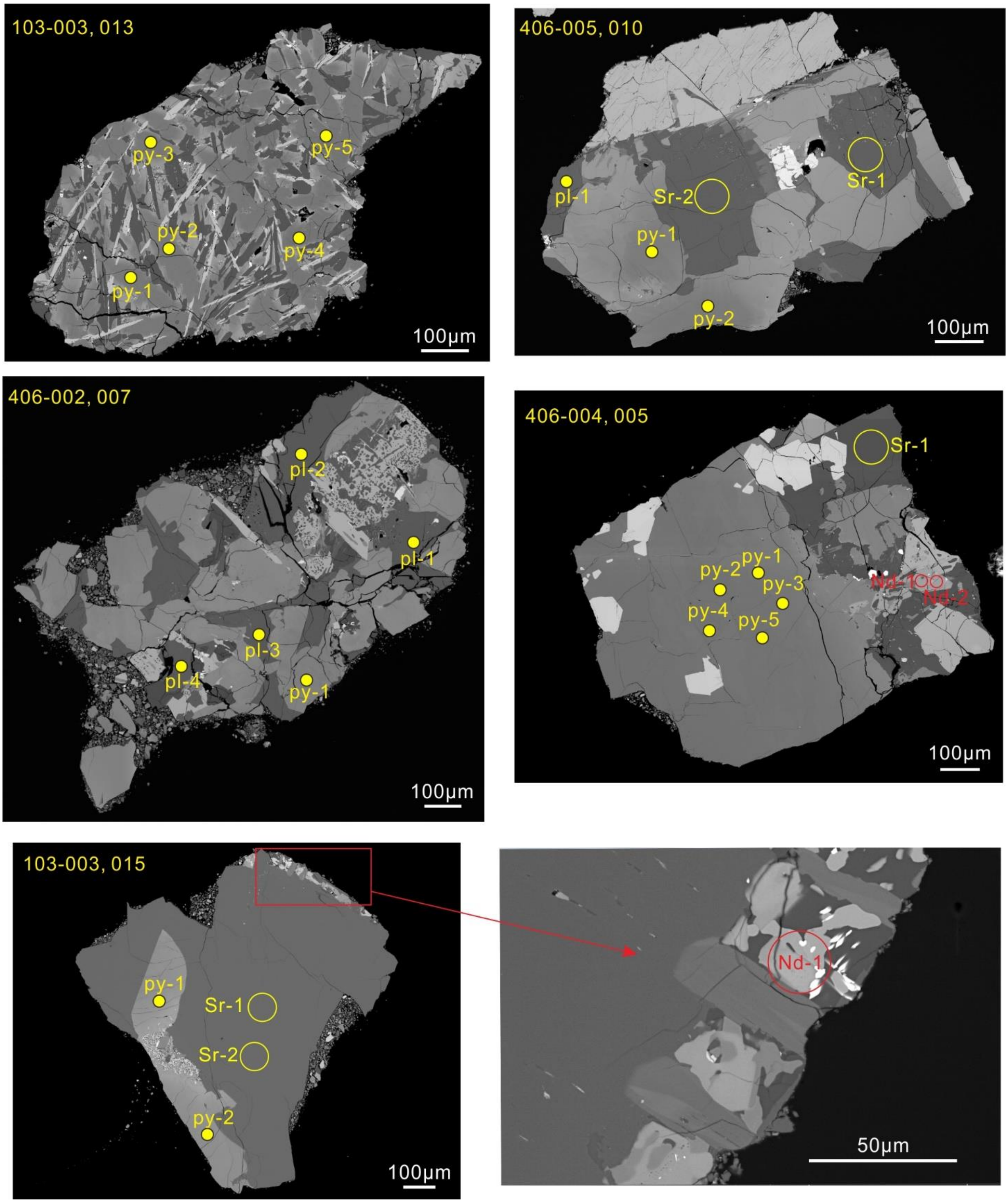

Figure S3. Backscatter Electron (BSE) images of the Chang'E-5 basalts (103-003,013; 406-005,010; 406-002,007; 406-004,005; 103-003,015). The black circles filled with yellow color, yellow circles, and red circles show the LA-(MC)-ICP-MS spots for trace elements, Sr, and Nd isotopes, respectively.
